# Exploratory analysis of immune checkpoint receptor expression by circulating T cells and tumor specimens in patients receiving neo-adjuvant chemotherapy for operable breast cancer

**DOI:** 10.1186/s12885-020-06949-4

**Published:** 2020-05-19

**Authors:** Robert Wesolowski, Andrew Stiff, Dionisia Quiroga, Christopher McQuinn, Zaibo Li, Hiroaki Nitta, Himanshu Savardekar, Brooke Benner, Bhuvaneswari Ramaswamy, Maryam Lustberg, Rachel M. Layman, Erin Macrae, Mahmoud Kassem, Nicole Williams, Sagar Sardesai, Jeffrey VanDeusen, Daniel Stover, Mathew Cherian, Thomas A. Mace, Lianbo Yu, Megan Duggan, William E. Carson

**Affiliations:** 1 The Ohio State University Comprehensive Cancer Center, The Ohio State University, 410 W 12th Avenue, Columbus, OH 43210 USA; 2 Department of Internal Medicine, Division of Medical Oncology, The Ohio State University, Starling Loving Hall, 320 W10th Ave, Columbus, OH 43210 USA; 3 Division of Medical Oncology, James Cancer Hospital and the Ohio State University Comprehensive Cancer Center, 1800 Cannon Drive, 1250 Lincoln Tower, Columbus, OH 43210 USA; 4 Department of Surgery, The Ohio State University, 410 W 10th Ave, N911 Doan Hall, Columbus, OH 43210 USA; 5 Department of Pathology, The Ohio State University, 410 W 10th Ave, N337B Doan Hall, Columbus, OH 43210 USA; 6 Roche Tissue Diagnostics, 1910 E. Innovation Park Drive, Tucson, AZ 85744 USA; 7 Center for Biostatistics, The Ohio State University, 2012 Kenny Rd, Columbus, OH 43221 USA

**Keywords:** Breast cancer, Tumor-infiltrating lymphocytes, CD8+ T cells, Immune checkpoint receptors

## Abstract

**Background:**

While combinations of immune checkpoint (ICP) inhibitors and neo-adjuvant chemotherapy (NAC) have begun testing in patients with breast cancer (BC), the effects of chemotherapy on ICP expression in circulating T cells and within the tumor microenvironment are still unclear. This information could help with the design of future clinical trials by permitting the selection of the most appropriate ICP inhibitors for incorporation into NAC.

**Methods:**

Peripheral blood samples and/or tumor specimens before and after NAC were obtained from 24 women with operable BC. The expression of CTLA4, PD-1, Lag3, OX40, and Tim3 on circulating T lymphocytes before and at the end of NAC were measured using flow cytometry. Furthermore, using multi-color immunohistochemistry (IHC), the expression of immune checkpoint molecules by stromal tumor-infiltrating lymphocytes (TILs), CD8+ T cells, and tumor cells was determined before and after NAC. Differences in the percentage of CD4+ and CD8+ T cells expressing various checkpoint receptors were determined by a paired Student’s t-test.

**Results:**

This analysis showed decreased ICP expression by circulating CD4+ T cells after NAC, including significant decreases in CTLA4, Lag3, OX40, and PD-1 (all *p* values < 0.01). In comparison, circulating CD8+ T cells showed a significant increase in CTLA4, Lag3, and OX40 (all *p* values < 0.01). Within tumor samples, TILs, CD8+ T cells, and PD-L1/PD-1 expression decreased after NAC. Additionally, fewer tumor specimens were considered to be PD-L1/PD-1 positive post-NAC as compared to pre-NAC biopsy samples using a cutoff of 1% expression.

**Conclusions:**

This work revealed that NAC treatment can substantially downregulate CD4+ and upregulate CD8+ T cell ICP expression as well as deplete the amount of TILs and CD8+ T cells found in breast tumor samples. These findings provide a starting point to study the biological significance of these changes in BC patients.

**Trial registration:**

NCT04022616.

## Background

Breast cancer (BC) is the most common malignancy in women, with over 1.3 million cases worldwide and 240,000 cases in the United States annually [1–3]. Approximately 93% of all newly diagnosed cases of BC in the United States are operable, but many patients require systemic chemotherapy in order to decrease the risk of locoregional and systemic recurrence [4]. Recently, there has been an increase in the use of neoadjuvant chemotherapy (NAC), especially for patients with triple-negative (TNBC) and human epidermal growth factor receptor 2 (HER2) + disease [5]. Randomized, controlled, prospective studies that compared NAC with adjuvant chemotherapy have shown that patient survival is similar between these two approaches [6, 7]. However, NAC offers several advantages over adjuvant chemotherapy, including the ability to increase the rate of breast conservation and to monitor for chemotherapy response [5, 8]. Notably, pathologic complete response (pCR) following NAC has emerged as a reliable surrogate marker of improved disease free survival (DFS) and overall survival (OS), especially in patients with TNBC and hormone receptor (HR)−/HER2+ disease [9].

Several studies have shown that the presence of tumor-infiltrating lymphocytes (TILs) is associated with higher rates of pCR to NAC [1, 10–12]. Furthermore, many studies have revealed that TIL levels are predictive of response to NAC and that for individuals with TNBC and HER2+ BC, TIL levels were positively associated with a survival benefit [10, 11, 13–15]. These data suggest that the immune system may play a role in controlling breast cancer and that the cytotoxic agents used in NAC may function in part through modulation of the immune system. This observation opens up the possibility that immune therapies could be incorporated into NAC for BC. Several such approaches are currently under investigation in multiple clinical trials [16].

In the metastatic setting, the IMpassion130 trial showed a 7 month improvement in OS when the PD-L1 inhibitor atezolizumab was added to nab-paclitaxel chemotherapy in the front line setting for patients with TNBC and positive expression of PD-L1 on the immune cells within the tumor microenvironment [17, 18]. Similarly, results from the Keynote-522 trial have shown that addition of an immune checkpoint (ICP) inhibitor to standard BC NAC can improve the rate of pCR in TNBC patients [19]. Other studies that combine ICP inhibitors and NAC backbones are currently ongoing. For example, study NCI10013 adds atezolizumab to carboplatin and paclitaxel [20] and study NCT03289819 tests the addition of the PD-1 inhibitor pembrolizumab to neo-adjuvant nab-paclitaxel followed by epirubicin and cyclophosphamide.

Thus far, only antibodies targeting PD-1, PD-L1, and CTLA4 have received FDA approval for the treatment of cancer. However, it is likely that drugs targeting additional ICPs, such as Tim3, Lag3, and OX40, could be approved in the future [21, 22]. Tim3 is an inhibitory receptor that has been found to inhibit Th1 T cell responses, and there are several antibodies targeting Tim3 in development [21]. Lag3 is another checkpoint receptor expressed by regulatory T cells and TILs that has been shown to dampen anti-tumor immune responses [21]. Finally, OX40 is a co-stimulatory molecule expressed by activated CD4+ and CD8+ T cells [21]. Agonists of OX40 can induce T cell proliferation and expansion [23].

In order to effectively incorporate immune therapy into NAC for BC, it will be important to understand the changes that occur in the expression of ICP proteins during NAC, both in circulating T cells and within the tumor. Thus, the goal of this study was to evaluate the changes that occur in the expression of PD-1, CTLA4, Tim3, Lag3, and OX40 by circulating CD4+ and CD8+ T cells in response to NAC. Levels of stromal TILs and tumor PD-1/PD-L1 expression were also evaluated in BC patients receiving NAC.

## Methods

### Study design

Specimens for this analysis were obtained under an IRB-approved, single-arm correlative study that was conducted at The Ohio State University Comprehensive Cancer Center between May 2012 and March 2014 (IRB protocol No. 2010C0036). Eligible patients included adult women (≥18 years old) with biopsy proven, non-metastatic BC who, in the opinion of the treating physician, were suitable for NAC. Exclusion criteria were the presence of inoperable BC or receipt of chemotherapy for breast cancer prior to study enrollment. All patients were required to sign an IRB-approved informed consent form prior to enrollment.

### Neo-adjuvant chemotherapy

Eligible participants received intravenous NAC as determined by the treating physician. The chemotherapy regimens employed in this study have previously been described and are listed in Additional File 1 [2]. Briefly, the majority of patients received 4 cycles of doxorubicin and cyclophosphamide given every 2 weeks at standard doses, followed by either 12 treatments of paclitaxel given weekly or 4 cycles of dose-dense paclitaxel given every 2 weeks. For patients with HER2+ BC, trastuzumab was administered alone or in combination with pertuzumab along with the paclitaxel. For all chemotherapy regimens, dexamethasone was utilized as an anti-emetic agent (frequency and timing detailed in Additional File 1). Peripheral blood samples were all obtained prior to administration of chemotherapy. All blood draws were performed 7 days or more from the last dose of dexamethasone. Residual post-NAC tumor samples were obtained three or more weeks after the last dose of dexamethasone.

### Sample collection and procurement

Peripheral blood was collected prior to the first and last cycle of NAC for this study. Peripheral blood mononuclear cells (PBMCs) were isolated from peripheral venous blood via density gradient centrifugation with Ficoll-Paque (Amersham Pharmacia Biotech, Uppsala, Sweden), as previously described [24, 25]. PBMCs were cryopreserved and stored at − 80 °C until 1 × 10^6^ PBMCs from all compared samples could be concurrently thawed and analyzed by flow cytometry. Assessment of ICP expression on CD4+ and CD8+ T cells was performed at baseline and at the time of the last chemotherapy treatment. Archived formalin-fixed, paraffin-embedded pre-NAC biopsies and post-NAC resection specimens were retrieved for analysis of TILs, CD8+ T cells and PD-L1 and PD-1 expression.

### Flow cytometry for expression of ICPs on circulating T cells

PBMCs were stained with fluorescent antibodies to CD4, CD8, CTLA4, PD-1, Lag3, OX40, and Tim3. Specific antibodies and fluorophores were as follows: CD4 FITC, CD8 APC, PD-1 PE, Lag3 PE, Tim3 PE, CTLA4 PE, OX40 PE. To perform flow cytometry compensation and verify fluorescent antibody efficacy, the AbC Total Antibody Compensation Bead Kit (Thermo Fischer Scientific, Waltham, MA) was utilized according to manufacturer’s instructions to determine positive and negative cell populations. Gating on CD4+ cells identified T helper lymphocytes and gating on CD8+ cells identified cytotoxic T lymphocytes. CD4+ and CD8+ T cells were subsequently analyzed separately for expression of CTLA4, PD-1, Lag3, OX40, and Tim3. All samples were run on a BD LSR-II flow cytometer and data was analyzed with FlowJo software (Tree Star, Inc.). Differences in the expression of ICP receptors before and after NAC were determined by comparing the percentage of CD4+ or CD8+ T cells expressing a given ICP.

### Analysis of tumor immune infiltrate

A multi-color immunohistochemistry (IHC) multiplex assay simultaneously detecting PD-1, PD-L1, and CD8 expressing cells (Roche Tissue Diagnostics) was performed on whole sections from formalin-fixed, paraffin-embedded pre-NAC biopsies or post-NAC resected tumor specimens. In this assay, PD-L1 staining is brown, PD-1 staining is red, and CD8 staining is green. Membranous staining was considered to be specific. A cut off of ≥1% was employed to define PD-1 or PD-L1 positive expression, as this was previously determined to be an appropriate measure of PD-L1 positivity and associated with improved outcomes for the addition of PD-L1 inhibitors to chemotherapy in several clinical trials [17, 26]. PD-L1 positive expression in the tumor is reported as the percentage of PD-L1 positive tumor cells amongst total tumor cells. Similarly, within the stroma, the amount of PD-L1 positive stromal/immune cells is reported as the percentage of PD-L1 positive stromal/immune cells amongst total stromal/immune cells. Total PD-L1 positive cells are reported as the total percentage of PD-L1 positive tumor and stromal/immune cells amongst total tumor and stromal/immune cells. The amount of CD8+ T cells within the tumor, stroma, and the total sample was calculated by comparing CD8+ immune cells to total immune cells within tumor area, stromal area, and entire area respectively. TILs were identified on hematoxylin and eosin stained whole sections and defined as the percent of stromal area within/surrounding tumor containing infiltrating lymphocytes compared to the total area. Analysis of tumor specimens was performed by an experienced pathologist specializing in BC and tumor microenvironment (ZL).

### Statistical analysis

Statistical differences between treatment groups were determined using paired (when comparing pre- and post-NAC samples) and unpaired (when comparing between tumor subtypes) Student’s t-tests. On presented graphs, bars represent group means and each pair of connecting circles signify individual patient values pre- and post-NAC.

## Results

### Patient characteristics

Twenty-four women with operable BC were enrolled in this study. Two patients did not complete all of the required blood draws and were therefore only included in the tumor specimen analysis. Patient characteristics are summarized in Table 1. The median patient age was 48 years (range 32–70). All patients were Eastern Cooperative Oncology Group (ECOG) performance status of 0 or 1, indicative of all patients being completely ambulatory. The majority of patients were Caucasian (*n* = 17) and pre-menopausal (*n* = 15). Eleven patients had TNBC, eight had HR+/HER2- BC, three patients had HR−/HER2+ BC, and two patients had HR+/HER2+ BC. Only one patient had stage I disease, while 20 and 3 patients had stage II and III BC, respectively. All 24 patients had invasive ductal carcinoma as the tumor histology. These characteristics are felt to be representative of a typical patient population that is offered NAC [27]. The overall rate of pCR, which is defined as no pathologic evidence of residual invasive cancer in the breast and sampled regional lymph nodes, was 41.7% (45.5% in patients with TNBC, 37.5% for patients with HR+/HER- BC, 66.7% in patients with HR−/HER2+ BC, and 0% for patients with HR+ HER2+ BC). The rates of pCR and residual cancer burden indexes [28] by NAC regimen are reported in Additional File 1. The surgical management of the patients’ BC following NAC are detailed in Additional File 2.

**Table 1:** Patient demographics

Characteristic		N	pCR rate
All Patients		26	46.2%
Race	White	19	52.6%
	African American	6	16.7%
	Hispanic	1	100%
Age (years)	Median	48	
	Range	32–70	
ECOG performance status	0	21	47.6%
	1	5	40%
Menopause status	Pre-menopausal	16	37.5%
	Post-menopausal	7	85.7%
	Unknown	3	0%
Tumor size (cm)	Median	2.8	
	Range	0.6–8.7	
Clinical node stage	0	14	35.7%
	1	11	54.5%
	2	1	100%
Clinical stage	IA	1	100%
	IIA	14	35.7%
	IIB	8	50%
	IIIA	3	66.7%
Grade	1	0	
	2	7	0%
	3	17	63.2%
Receptor status	HR+ and HER-2-	8	37.5%
	HR+ and HER-2+	3	33.3%
	HR- and HER-2+	4	75%
	Triple Negative	11	45.5%

### Circulating CD4+ and CD8+ T cell expression of ICP receptors

**Fig. 1:**
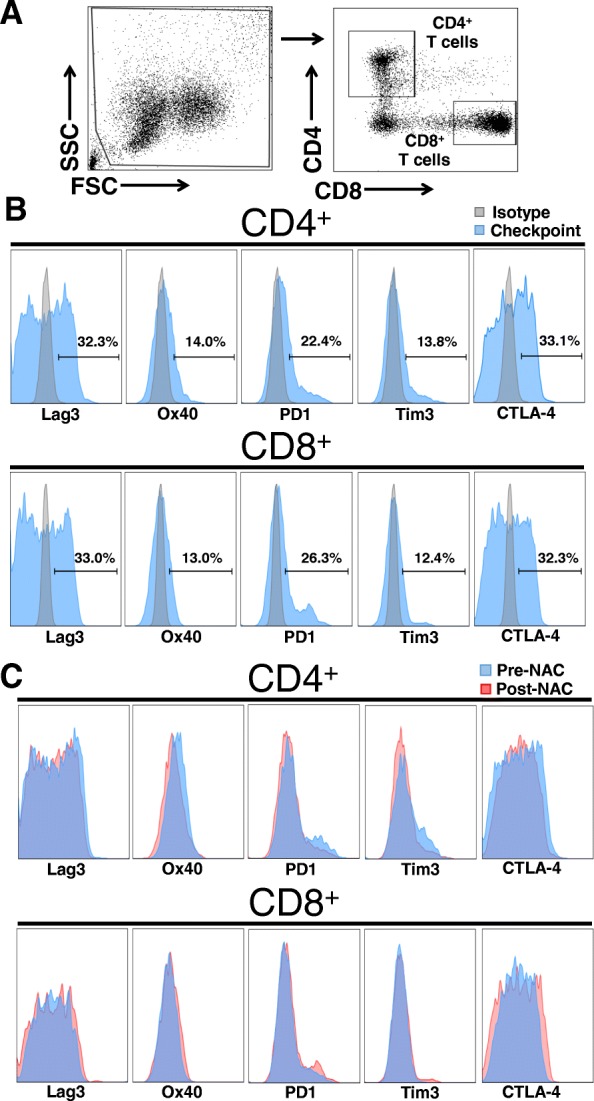
Representative flow cytometry plots demonstrating gating strategy to identify CD4+ and CD8+ T cells as well as expression of various immune checkpoint receptors. (**a**) Representative scatter plots to show gating for CD4+ and CD8+ T cells. (**b**) Histograms of isotype controls are shown in gray and checkpoint receptor (CTLA4, Lag3, OX40, PD-1, and Tim3) expressions are shown in blue. Positive measurement of each checkpoint receptor is demonstrated within brackets. (**c**) Representative histograms for pre-NAC (blue) and post-NAC (red) checkpoint receptor expression are shown

**Fig. 2:**
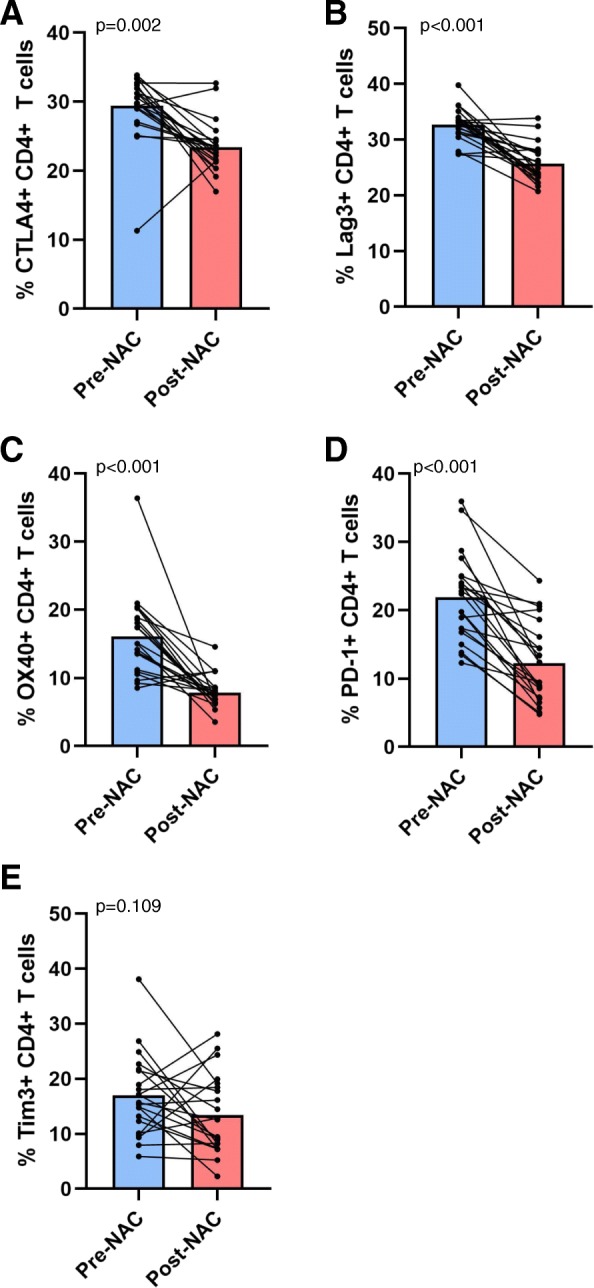
Changes in the frequency of CD4+ T cells expressing immune checkpoint receptors. Pre- and post-NAC levels of specified CD4+ T cells are shown with each pair of connecting circles representing individual patient levels of (**a**) CTLA4+, (**b**) Lag3+, (**c**) OX40+, (**d**) PD-1+, or (**e**) Tim3+ cells at these time points. Bars are representative of mean ICP levels. Paired Student’s t test was used to compare pre- and post-NAC levels of specified T cell subsets

**Fig. 3:**
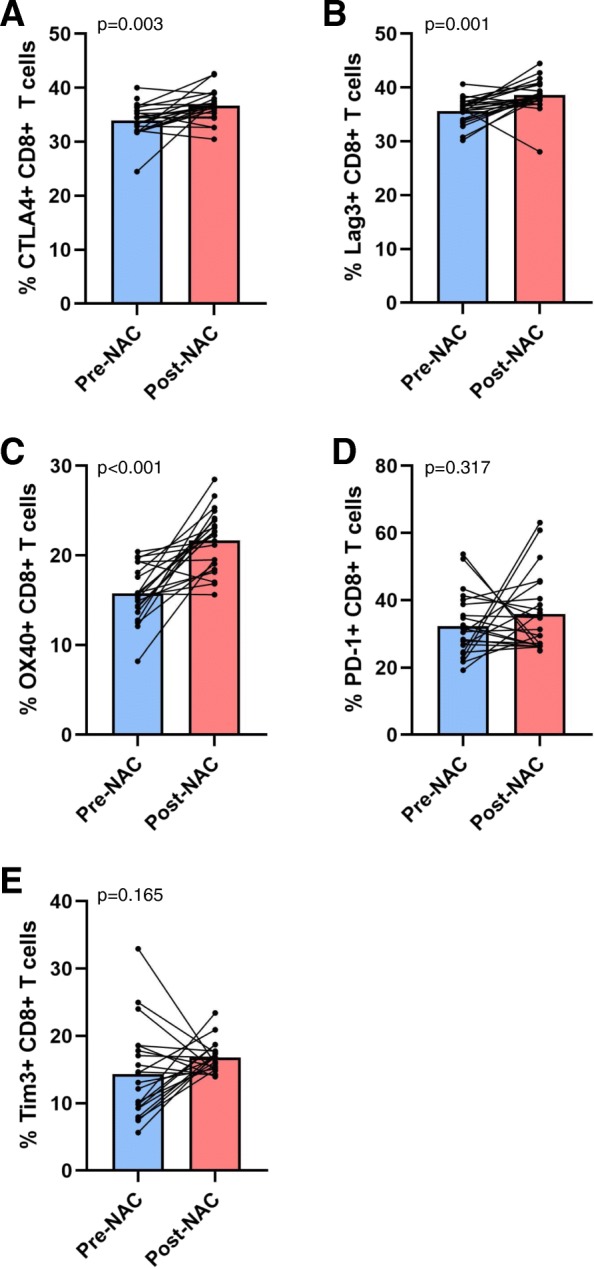
Changes in the frequency of CD8+ T cells expressing immune checkpoint receptors. Pre- and post-NAC levels of specified CD8+ T cells are shown with each pair of connecting circles representing individual patient levels of (**a**) CTLA4+, (**b**) Lag3+, (**c**) OX40+, (**d**) PD-1+, or (**e**) Tim3+ cells at these time points. Bars are representative of mean ICP levels. Paired Student’s t test was used to compare pre- and post-NAC levels of specified T cell subsets

Flow cytometry was used to assess the overall frequency of peripheral blood CD4+ and CD8+ T cells and their expression of ICP receptors (CTLA4, Lag3, OX40, PD-1, and Tim3) in 22 patients (see Fig. 1 for representative flow cytometry plots and gating strategy) and individual patient expression levels pre- and post-NAC were compared in a paired t-test. Following NAC, there was found to be a significant decrease in the percentage of CD4+ T cells expressing CTLA4 (29.4% vs. 23.4%, *p* < 0.01), Lag3 (32.7% vs. 25.7%, *p* < 0.001), OX40 (16.1% vs. 7.9%, *p* < 0.001), and PD-1 (21.8% vs. 12.2%, *p* < 0.001) (Fig. 2a-d). Additionally, there was a numerical trend toward fewer CD4+ T cells expressing Tim3 which did not reach statistical significance (17.0% vs. 13.5%, *p* = 0.109) (Fig. 2e). In contrast, there was a significant increase in the percentage of CD8+ T cells expressing CTLA4 (34.0% vs. 36.7%, *p* < 0.01), Lag3 (35.6% vs. 38.6%, *p* = 0.001), and OX40 (15.7% vs. 21.7%, *p* < 0.001) after NAC (Fig. 3a-c). There was also a trend towards increased PD-1 (32.2% vs. 35.9%, *p* = 0.317) and Tim3 expression (14.4% vs. 16.8%, *p* = 0.165) on CD8+ T cells following NAC, but neither of these values reached statistical significance (Fig. 3d-e).

Differences in ICP expression dependent upon breast tumor subtype were also examined. In Additional File 3, TNBC patients’ peripheral blood CD4+ and CD8+ T cell expression of ICPs were compared to patients with other breast cancer subtypes. In this analysis, the only statistically significant difference seen was greater pre-NAC CD8+ T cell Tim3 expression in TNBC patients over patients with other breast cancer subtypes (*p* < 0.05). HR+ and HR- patient levels of ICP expression were also compared in Additional File 4. In accordance with the prior analysis, pre-NAC CD8+ T cell Tim3 expression was lower in HR+ blood specimens than HR- samples (*p* < 0.01). No other statistically relevant differences were seen.

### Tumor infiltrating lymphocytes in tumor samples before and after NAC

**Fig. 4:**
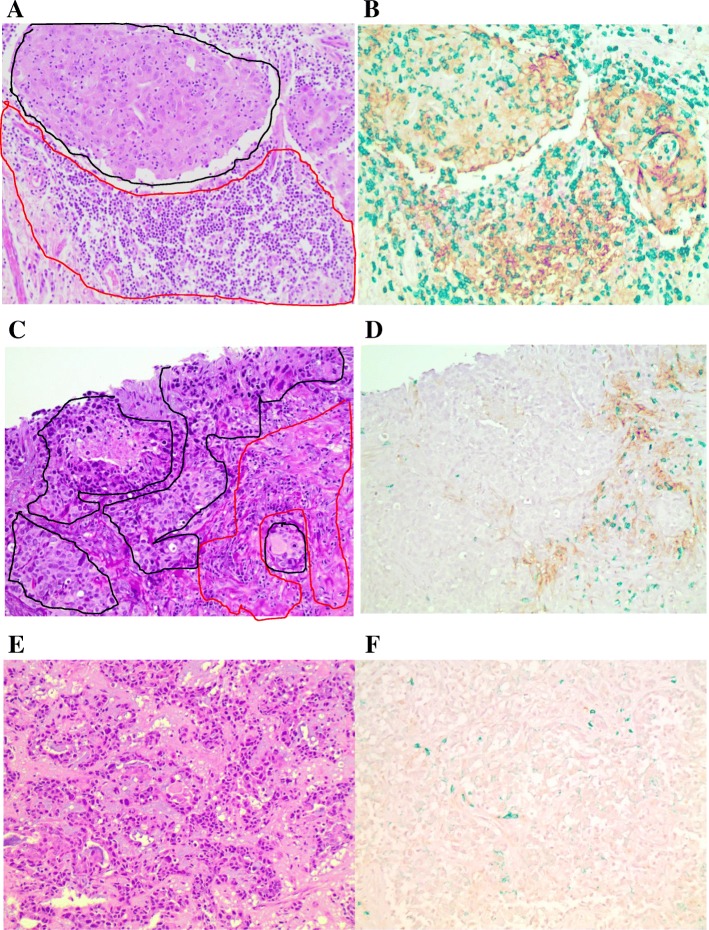
Representative images for immuno-histochemical (IHC) analysis of CD8, PD-L1, and PD-1 expression. (**a**) Representative H&E staining showing a portion of tumor outlined in black and a portion of stroma outlined in red. (**b**) Representative image showing multicolor IHC staining for all three markers: PD-L1, PD-1, and CD8. Brown staining identifies PD-L1 expression, red identifies PD-1 expression, and green represents CD8 expression. Percentage of cells expressing various markers was determined as the area expressing the marker divided by the total tumor area, total stromal area, or tumor and stromal area together. (**c**) Additional representative H&E staining showing tumor outlined in black and stroma outlined in red. (**d**) Representative multicolor IHC staining showing PD-L1 (brown) and CD8 (green) staining in the stroma only with no staining in the tumor. (**e**) Representative H&E staining. (**f**) Representative multicolor IHC staining showing only rare CD8 (green) staining within the stroma and no PD-L1 (brown) or PD-1 (red) staining

Biopsy specimens prior to NAC were available from 6 patients, and resection samples after NAC were available from 17 patients. A representative H&E slide demonstrating the areas defined as tumor (black) and stroma (red) for TIL determination is shown in Fig. 4a. In the pre-NAC samples, an average of 29.8% of the stroma contained TILs, compared to 24.9% TILs in the stroma of post-NAC samples (Table 2).

**Table 2:** Changes in TILs and CD8+ T cells following NAC

	Pre-NAC cohort (***n*** = 6)	Post-NAC cohort (***n*** = 17)
**Percentage of TILs**		
Stromal TILs	29.8% (1–80%)	24.9% (2–70%)
**Percentage of CD8+ T cells**		
Overall CD8+ T cells	18.3% (0.5–60%)	15.7% (1–50%)
Stromal CD8+ T cells	24.6% (1–70%)	21.2% (1–60%)
Intratumoral CD8+ T cells	12.0% (0–50%)	7.9% (0–40%)

Values are denoted as means with ranges in the parentheses

In the pre-NAC group, the range of stromal area containing TILs was 1–80%, with 3/6 samples having more than 10% and 2/6 samples having greater than 50% TILs. In the post-NAC group, the range of TILs was similar at 2–70%, with 11/17 samples having greater than 10% and 3/17 samples having greater than 50%. It should be noted that of the patients with available pre-NAC specimens, only one (16.7%) ended up having pCR. Of the patients with available post-NAC specimens, three (17.6%) had pCR. No analysis for statistical significance was performed due to limited sample size.

### Frequency and location of CD8+ T cells in tumor samples before and after NAC

To evaluate changes in CD8+ T cell localization, the percentage of stromal or tumor areas containing CD8+ cells was calculated by dividing the area containing CD8+ cells by the total area in either the stroma or the tumor. In addition, the percentage of CD8+ cells within the entire sample was determined by combining stromal and tumor analysis for each sample (i.e. tumor and stroma together). Representative images of the IHC analysis of CD8+ T cells are available in Fig. 4. In the stroma alone, an average of 24.6% of cells were CD8+ in the pre-NAC specimens, while an average of 21.2% of stromal cells were CD8+ following NAC. Within the tumor alone, an average of 12.0% of cells were CD8+ prior to NAC, and in the post-NAC samples, only 7.9% of cells were CD8+. In the pre-NAC samples, 18.3% (range 0.5–60%) of cells in the stroma and tumor combined were CD8+, while 15.7% (range 1–50%) in the post-NAC group were CD8+ (Table 2).

### PD-L1 and PD-1 expression in biopsy and residual tumor samples

PD-L1/PD-1 expression was evaluated in the pre-NAC (*n* = 6) and post-NAC (*n* = 17) samples, with PD-L1/PD-1 positive patients defined as having ≥1% of cells staining positively for PD-L1/PD-1. Representative images of the IHC analysis of PD-L1 and PD-1 are provided in Fig. 4. Among the pre-NAC samples, 4/6 patients (66.7%) were positive for overall PD-L1 expression (stroma and tumor together), 3/6 (50%) were positive for tumor PD-L1 expression, and 4/6 (66.7%) were positive for stromal PD-L1 expression. Among the post-NAC samples, 9/17 patients (52.9%) were positive for overall PD-L1 expression, 5/17 (29.4%) were positive for tumor PD-L1 expression, and 10/17 (58.8%) were positive for stromal PD-L1 expression. PD-1 expression was similarly evaluated in these tumor tissues. For PD-1 expression, 2/6 (33.3%) patients in the pre-NAC tumor specimens were positive for overall PD-1 expression, while 4/17 (23.5%) post-NAC specimens were positive. These results are summarized in Table 3. The intensity of PD-L1/PD-1 expression in the tumor, stroma, and overall cells are listed in Additional File 5.

**Table 3:** Number of patients with PD-L1/PD-1 positive tumors and stroma

	Pre-NAC cohort (***n*** = 6)	Post-NAC cohort (***n*** = 17)
Overall PD-L1+	4 (66.7%)	9 (52.9%)
Intratumoral PD-L1+	3 (50.0%)	5 (29.4%)
Stromal PD-L1+	4 (66.7%)	10 (58.8%)
Overall PD-1+	2 (33.3%)	4 (23.5%)

Values are denoted as the number of patients in each group with percentage of cohort that is PD-L1 or PD-1 positive in the parentheses

### Analysis by breast cancer subset and patients with paired samples

There were three pre-NAC specimens and nine post-NAC specimens available for analysis of samples from patients with TNBC (Additional File 6). Additionally, there were two pre-NAC specimens and seven post-NAC samples from patients with hormone receptor positive breast cancer that were obtainable for study (Additional File 7). Overall, the levels of TILs, CD8+ T cells, and PD-L1/PD-1 expression in both of these groups remained stable after NAC.

**Fig. 5:**
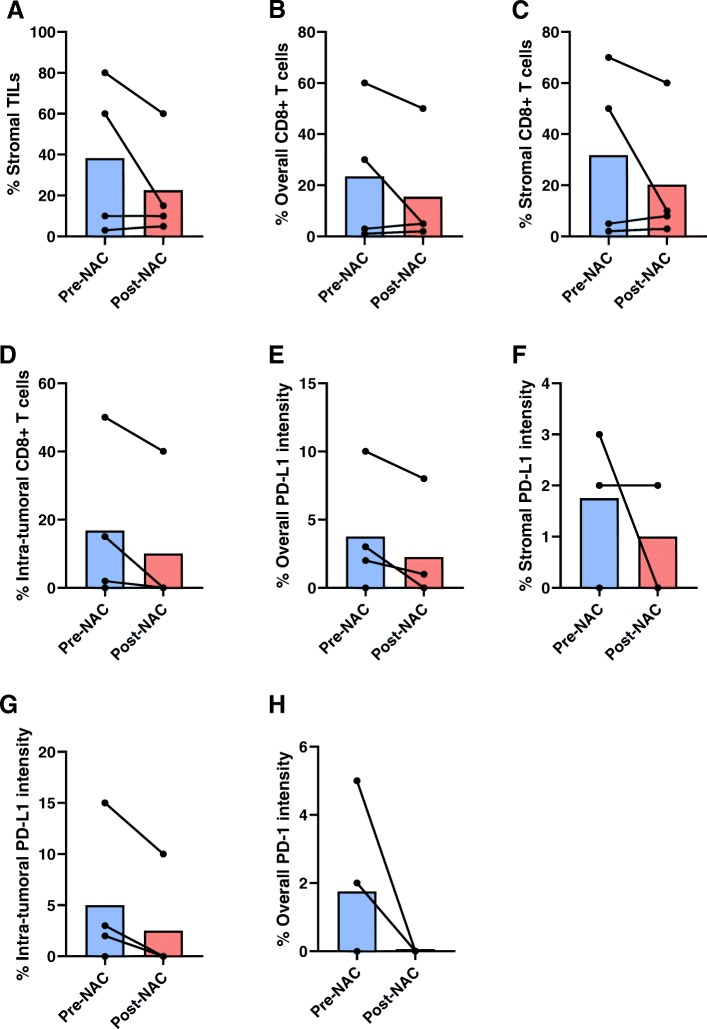
Percentage of TILs, CD8+ T cells, and PD-L1+/PD-1+ cells in patients with paired samples. Pre- and post-NAC levels of specified CD8+ T cells are shown with each pair of connecting circles representing individual patient levels of (**a**) stromal TILs, (**b**) overall CD8+ cells, (**c**) stromal CD8+ cells, (**d**) intra-tumoral CD8+ cells, (**e**) overall PD-L1 intensity, (**f**) stromal PD-L1 intensity, (**g**) intra-tumoral PD-L1 intensity, and (**h**) overall PD-1 intensity at these times points. *N* = 4 for each group, if a sample is not graphed it is due to values being 0

Four paired pre-NAC and post-NAC tissue samples were available for comparison and revealed amounts of TIL and CD8+ T cells, as well as PD-L1/PD-1 expression, to be mostly unchanged prior to and after NAC (Fig. 5). Since these patients by definition did not exhibit a pCR following neoadjuvant chemotherapy, it is not possible to interpret these paired results in the context of the full study population in which the pCR rate was 42%. However, visualization of individual patient levels of peripheral blood T cell ICP expression next to the same patient’s intra-tumoral PD-L1 intensity was completed (Additional File 8). Due to the small number of samples, no formal statistical analyses were performed to compare peripheral blood ICP levels to intra-tumoral levels of PD-L1 and no clear trends are seen on graphics.

## Discussion

NAC is an increasingly adopted treatment strategy for women with early-stage operable BC. Importantly, the development of new combinations of cytotoxic chemotherapy and ICP inhibitors in the neo-adjuvant setting may improve pCR rates, DFS, and OS. However, the influence of NAC on immune checkpoint expression has yet to be studied in a comprehensive manner. To address this issue, we present the results of a study that used flow cytometry and multi-color IHC to characterize expression of PD-1, CTLA4, Tim3, Lag3, and OX40 by circulating CD4 and CD8 T cells, as well as the level of TILs, infiltrating CD8+ T lymphocytes, and PD-1/PD-L1 expression within tumor samples obtained before and after NAC. Overall, this study found that NAC resulted in a decrease in checkpoint receptor expression (CTLA4, Lag3, OX40, and PD-1) by circulating CD4+ T helper lymphocytes. In contrast, when looking at CD8+ T cytotoxic lymphocytes, there was an increase in CTLA4, Lag3, and OX40 expression following NAC. Only expression of Tim3 was not statistically different between baseline and post- chemotherapy samples on circulating CD4+ and CD8+ T cells. Intratumorally, we observed that less of our samples were considered to be positive for the expression of either PD-L1 or PD-1 following NAC. The percentage of stromal TILs, CD8+ lymphocytes, and PD-L1 positivity in these patients decreased after NAC. In contrast, these values were relative stable between baseline and post-chemotherapy in the triple-negative tumors although the small sample size (*n* = 3 for pre-NAC baseline biopsy and *n* = 9 for post-NAC resection residual tumors) precluded formal statistical comparisons. Furthermore, the small sample size did not allow for the testing of the association between pCR rate and levels of stromal TILs, CD8+ lymphocytes, and PD-L1/PD-1 expression.

Several investigators have noted that the frequency of TILs is associated with increased rates of pCR. It has also been shown that TILs are associated with a survival benefit in patients with TNBC and HER2+ BC [13–15, 29]. Furthermore, several of the agents currently used in NAC regimens have been shown to modulate aspects of the immune system. For example, doxorubicin has been shown to promote antigen presentation by dendritic cells and help drive antigen-specific CD8+ T cell responses in mouse models [30–32]. Cyclophosphamide can stimulate natural killer cell anti-tumor responses, as well as promote macrophage recruitment to tumors and skew them towards an anti-tumor M1 like phenotype [33–36]. There are also several reports supporting the notion that administration of cyclophosphamide enhances the action of tumor-specific adoptive T cell therapy [37–39]. Finally, paclitaxel has been shown to promote the cytotoxicity of tumor-associated macrophages, increase natural killer cell activity, and stimulate tumor specific CD8 T cell responses [40–42]. These findings suggest that incorporation of therapies aimed at leveraging the immune system against BC could lead to more effective NAC regimens and improve the rate of pCR. It should also be pointed out that all patients in this study received intravenous dexamethasone as a standard pre-chemotherapy medication to prevent nausea and vomiting during the anthracycline portion of chemotherapy and/or to minimize the risk of severe hypersensitivity reactions prior to paclitaxel administration. While the impact of episodic steroid use is unclear, it is possible that any use of steroids may also affect the immune tumor microenvironment.

To date, the knowldege about influence of NAC on the expression of targetable checkpoint receptors has been limited. In order to optimally incorporate immune therapies into NAC regimens it will be important to understand how these agents affect the host immune system as well as the ability of tumor cells to impact infiltrating T cells. Recently, a report published by Pelekanou et al. found that following NAC use in breast cancer cases there was a decrease in the frequency of TILs, while PD-L1 expression was relatively stable [1, 43]. These results are consistent with the present analysis of pre- and post-treatment tumor specimens except that this study found a decrease in the PD-L1 expression in residual tumors following NAC. Furthermore, Pelekanou and colleagues showed that higher pre-treatment levels of TILs and PD-L1 expression were significantly associated with higher pCR rates [1]. These findings provide information that can be useful for incorporating immune therapies into NAC regimens for BC.

The current work helps expand on these findings by determining the expression of targetable checkpoint receptors on circulating CD4+ and CD8+ T cells before and after NAC. This analysis revealed a significant decrease in the frequency of circulating CD4+ T cell expressing CTLA4, Lag3, PD-1, and OX40 following NAC. In contrast, the frequency of CD8+ T cells expressing CTLA4, Lag3, and OX40 increased following NAC. The reason for the dichotomous change in the frequency of CD4+ and CD8+ T cells expressing checkpoint receptors is unclear. However, this effect could be driven by differences in the activation status of circulating CD4+ and CD8+ T cells after NAC or differences in the effect of chemotherapy agents on cytokine production by the T cell subsets. The decreased expression of the co-stimulatory molecule OX40 by CD4 T cells and its increase in CD8 T cells makes it an intriguing target as well.

The present study has several limitations that should be noted. First, the study was a single institutional experience and was limited by a small sample size in both the analysis of tumor specimens and circulating T cells. Also, the high rate of pCR contributed to the issue of not having substantial post-surgical samples. Furthermore, only four patients had paired tumor samples since many patients enrolled in the study had their biopsy performed at an outside institution and thus these samples were unavailable for review. Additional Files 5-7 document the recorded pre- and post-NAC changes in stromal TILs, CD8+ T cells, and PD-L1/PD-1 expression. Due to the small sample size, a meaningful statistical analysis of the correlation between pCR and TIL/ICP levels would not be possible. Nevertheless, these findings should serve as stimulus to investigate these changes in larger patient cohorts.

## Conclusions

In conclusion, this study shows that NAC use results in significant but opposite changes in the expression of ICP proteins by circulating CD4+ and CD8+ T cells in BC patients. In addition, the few tumor samples available post-NAC treatment appeared to have smaller frequencies of stromal TILs and intratumoral CD8+ T cells. Also, fewer of these post-NAC tumor samples were positive for PD-L1 or PD-1 following NAC To our knowledge, this study is the first to systematically assess peripheral blood expression of various ICPs together with changes in tumor immune infiltrates in women with non-metastatic BC. Understanding the effect of NAC on circulating and tumor-infiltrating immune cells will be important for optimally incorporating immune therapies into the NAC setting for BC. Furthermore, this work and that done by others serves as important data for the initiation of further studies to understand the mechanism and biological significance of these immunologic changes.

## Supplementary information

**Additional file 1.** Neo-adjuvant chemotherapy regimens. Table of the various neo-adjuvant chemotherapy regimens received by the patients in this study. N denotes the number of patients in each group. pCR represents the number (and percentage) of patients in each group with a pathologic complete response. RCB represents the median residual cancer burden score (and ranges) in each group.

**Additional file 2.** Surgical management. Table of the various surgical procedures received by the patients in this study. N denotes the number of patients in each group.

**Additional file 3.** ICP expression differences between TNBC patients and other breast cancer subtypes. Pre- and post-NAC levels of CD4+ and CD8+ T cell ICP expression were compared between the TNBC patients and other breast cancer subtype patients. Unpaired Student’s t-test was used to compare these groups. A green box indicates a statistically significant difference between TNBC and other breast cancer subtypes’ ICP expression.

**Additional file 4.** ICP expression differences between HR positive and HR negative breast cancer patients. Pre- and post-NAC levels of CD4+ and CD8+ T cell ICP expression were compared between the HR+ and HR- breast cancer patients. Unpaired Student’s t-test was used to compare these groups. A green box indicates a statistically significant difference between HR+ and HR- breast cancer patients’ ICP expression.

**Additional file 5.** Intensity of PD-L1 and PD-1 expression. Table of intensity of tissue staining for PD-L1 and PD-1. Values are denoted as means with ranges in the parentheses.

**Additional file 6.** Percentage of TILs, CD8+ T cells, and PD-L1+/PD-1+ cells in TNBC samples before and after NAC. Table of changes in TILs, CD8+ T cells, PD-L1 expression and PD-1 expression following NAC in samples from triple-negative breast cancer patients. There were three pre-NAC samples available and nine post-NAC samples available for analysis. Values are listed as means (and ranges) or the number of samples (and percentage of total group these represented). If samples stained < 1%, they were considered to have 0% expression for mean calculation. PD-L1 and PD-1 positivity was defined as ≥1% expression.

**Additional file 7.** Percentage of TILs, CD8+ T cells, and PD-L1+/PD-1+ cells in HR positive breast cancer samples before and after NAC. Table of changes in TILs, CD8+ T cells, PD-L1 expression and PD-1 expression following NAC in samples from HR positive breast cancer patients. There were two pre-NAC samples available and seven post-NAC samples available for analysis. Values are listed as means (and ranges) or the number of samples (and percentage of total group these represented). If samples stained < 1%, they were considered to have 0% expression for mean calculation. PD-L1 and PD-1 positivity was defined as ≥1% expression.

**Additional file 8.** Comparison of pre- and post-NAC ICP expression in peripheral blood T cells to intra-tumoral PD-L1 expression. Colored bars show individual values of (A) CTLA, (B) Lag3, (C) OX40, (D) PD-1, and (E) Tim3 expression in pre-NAC (solid pattern) and post-NAC (striped pattern) CD4+ (blue) and CD8+ (red) T cells. Black bars reveal pre-NAC (solid pattern) and post-NAC (striped pattern) levels of intra-tumoral PD-L1 intensity; values are also listed above bars).

## Data Availability

The datasets used and/or analyzed during the current study are available from the corresponding author upon a reasonable request.

## Abbreviations

BC: Breast cancer
ECOG: Eastern Cooperative Oncology Group
HER2: Human epidermal growth factor receptor 2
HR: Hormone receptor (estrogen and/or progesterone receptors)
ICP: Immune checkpoint
IHC: Immunohistochemistry
NAC: Neo-adjuvant chemotherapy
pCR: Pathological complete response
PBMC: Peripheral blood mononuclear cells
TILs: Tumor-infiltrating lymphocytes
TNBC: Triple-negative breast cancer
