# Supplementary material for: Exploratory analysis of immune checkpoint receptor expression by circulating T cells and tumor specimens in patients receiving neo-adjuvant chemotherapy for operable breast cancer
Source: BMC Cancer. 2020 May 19;20:445. doi: 10.1186/s12885-020-06949-4 (PMC7236344; doi:10.1186/s12885-020-06949-4)
Supplement: Supplementary file 1 — Additional file 1. Neo-adjuvant chemotherapy regimens. Table of the various neo-adjuvant chemotherapy regimens received by the patients in this study. N denotes the number of patients in each group. pCR represents the number (and percentage) of patients in each group with a pathologic complete response. RCB represents the median residual cancer burden score (and ranges) in each group. [file 12885_2020_6949_MOESM1_ESM.pptx]

## Slide 1
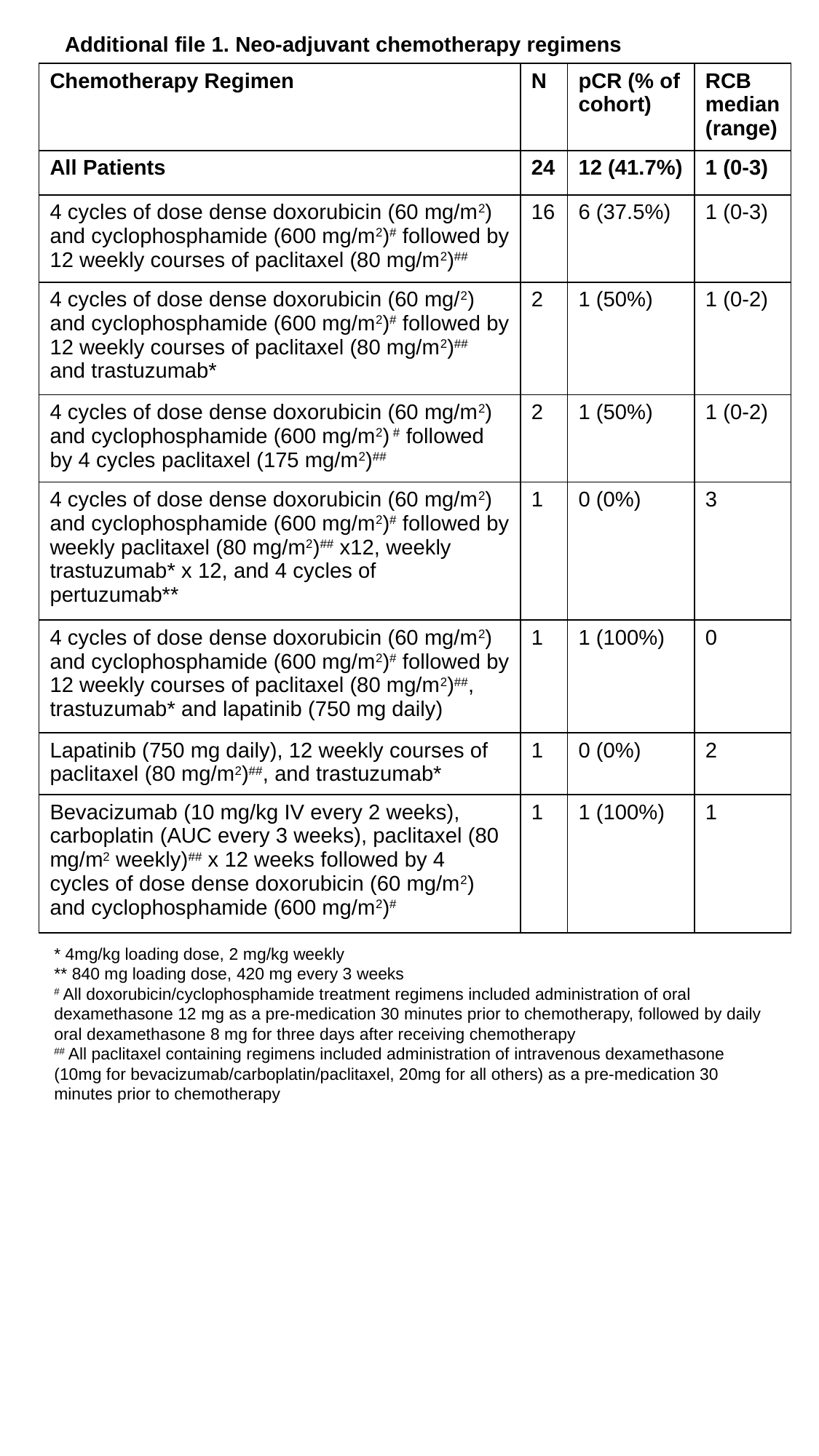

Additional file 1. Neo-adjuvant chemotherapy regimens
| Chemotherapy Regimen | N | pCR (% of cohort) | RCB median (range) |
| --- | --- | --- | --- |
| All Patients | 24 | 12 (41.7%) | 1 (0-3) |
| 4 cycles of dose dense doxorubicin (60 mg/m2) and cyclophosphamide (600 mg/m2)# followed by 12 weekly courses of paclitaxel (80 mg/m2)## | 16 | 6 (37.5%) | 1 (0-3) |
| 4 cycles of dose dense doxorubicin (60 mg/2) and cyclophosphamide (600 mg/m2)# followed by 12 weekly courses of paclitaxel (80 mg/m2)## and trastuzumab\* | 2 | 1 (50%) | 1 (0-2) |
| 4 cycles of dose dense doxorubicin (60 mg/m2) and cyclophosphamide (600 mg/m2) # followed by 4 cycles paclitaxel (175 mg/m2)## | 2 | 1 (50%) | 1 (0-2) |
| 4 cycles of dose dense doxorubicin (60 mg/m2) and cyclophosphamide (600 mg/m2)# followed by weekly paclitaxel (80 mg/m2)## x12, weekly trastuzumab\* x 12, and 4 cycles of pertuzumab\*\* | 1 | 0 (0%) | 3 |
| 4 cycles of dose dense doxorubicin (60 mg/m2) and cyclophosphamide (600 mg/m2)# followed by 12 weekly courses of paclitaxel (80 mg/m2)##, trastuzumab\* and lapatinib (750 mg daily) | 1 | 1 (100%) | 0 |
| Lapatinib (750 mg daily), 12 weekly courses of paclitaxel (80 mg/m2)##, and trastuzumab\* | 1 | 0 (0%) | 2 |
| Bevacizumab (10 mg/kg IV every 2 weeks), carboplatin (AUC every 3 weeks), paclitaxel (80 mg/m2 weekly)## x 12 weeks followed by 4 cycles of dose dense doxorubicin (60 mg/m2) and cyclophosphamide (600 mg/m2)# | 1 | 1 (100%) | 1 |
* 4mg/kg loading dose, 2 mg/kg weekly
** 840 mg loading dose, 420 mg every 3 weeks
# All doxorubicin/cyclophosphamide treatment regimens included administration of oral dexamethasone 12 mg as a pre-medication 30 minutes prior to chemotherapy, followed by daily oral dexamethasone 8 mg for three days after receiving chemotherapy
## All paclitaxel containing regimens included administration of intravenous dexamethasone (10mg for bevacizumab/carboplatin/paclitaxel, 20mg for all others) as a pre-medication 30 minutes prior to chemotherapy
