# Supplementary material for: Exploratory analysis of immune checkpoint receptor expression by circulating T cells and tumor specimens in patients receiving neo-adjuvant chemotherapy for operable breast cancer
Source: BMC Cancer. 2020 May 19;20:445. doi: 10.1186/s12885-020-06949-4 (PMC7236344; doi:10.1186/s12885-020-06949-4)
Supplement: Supplementary file 2 — Additional file 2. Surgical management. Table of the various surgical procedures received by the patients in this study. N denotes the number of patients in each group. [file 12885_2020_6949_MOESM2_ESM.pptx]

## Slide 1
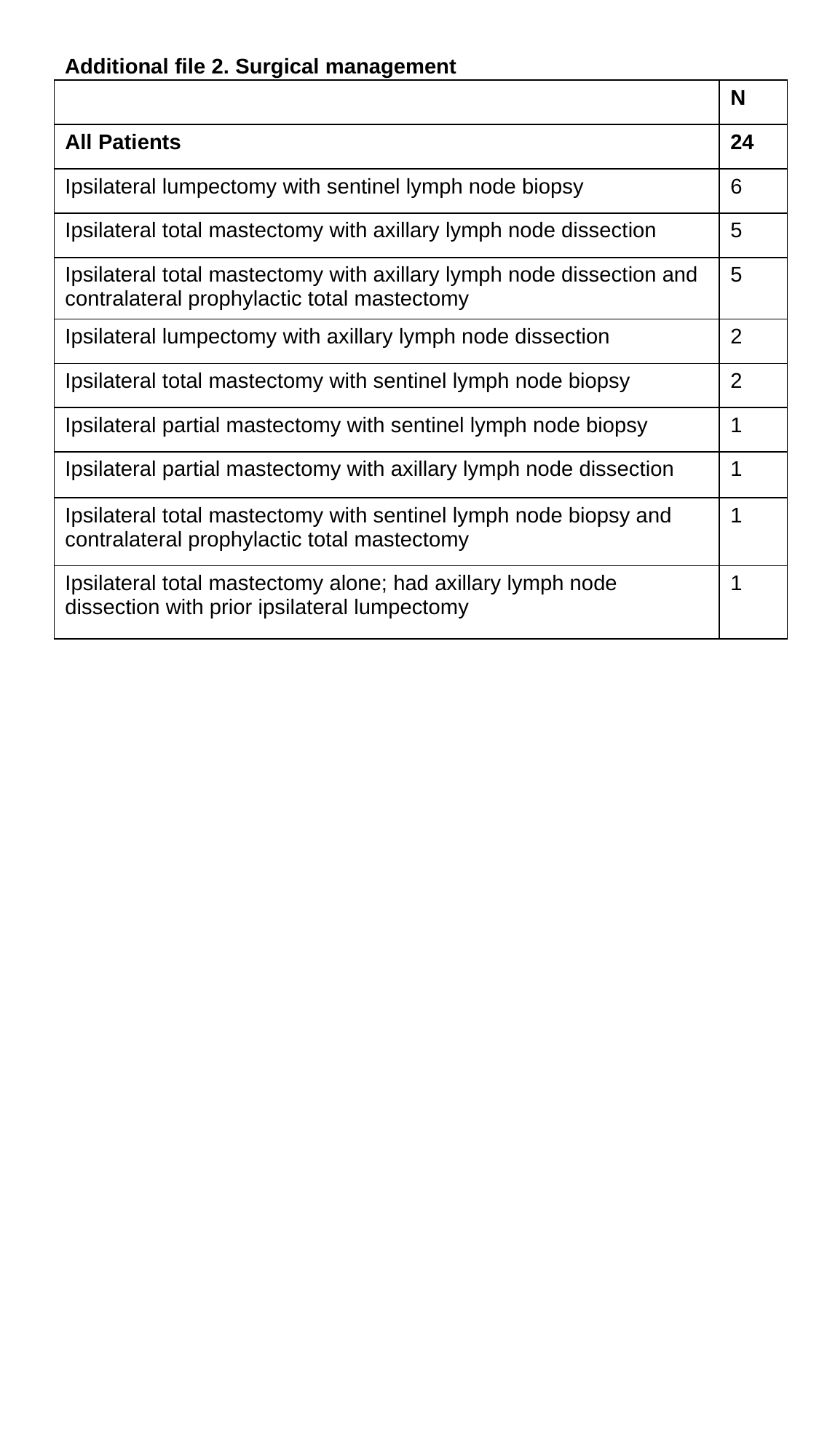

Additional file 2. Surgical management
| | N |
| --- | --- |
| All Patients | 24 |
| Ipsilateral lumpectomy with sentinel lymph node biopsy | 6 |
| Ipsilateral total mastectomy with axillary lymph node dissection | 5 |
| Ipsilateral total mastectomy with axillary lymph node dissection and contralateral prophylactic total mastectomy | 5 |
| Ipsilateral lumpectomy with axillary lymph node dissection | 2 |
| Ipsilateral total mastectomy with sentinel lymph node biopsy | 2 |
| Ipsilateral partial mastectomy with sentinel lymph node biopsy | 1 |
| Ipsilateral partial mastectomy with axillary lymph node dissection | 1 |
| Ipsilateral total mastectomy with sentinel lymph node biopsy and contralateral prophylactic total mastectomy | 1 |
| Ipsilateral total mastectomy alone; had axillary lymph node dissection with prior ipsilateral lumpectomy | 1 |
