# Supplementary material for: Exploratory analysis of immune checkpoint receptor expression by circulating T cells and tumor specimens in patients receiving neo-adjuvant chemotherapy for operable breast cancer
Source: BMC Cancer. 2020 May 19;20:445. doi: 10.1186/s12885-020-06949-4 (PMC7236344; doi:10.1186/s12885-020-06949-4)
Supplement: Supplementary file 3 — Additional file 3. ICP expression differences between TNBC patients and other breast cancer subtypes. Pre- and post-NAC levels of CD4+ and CD8+ T cell ICP expression were compared between the TNBC patients and other breast cancer subtype patients. Unpaired Student’s t-test was used to compare these groups. A green box indicates a statistically significant difference between TNBC and other breast cancer subtypes’ ICP expression. [file 12885_2020_6949_MOESM3_ESM.pptx]

## Slide 1
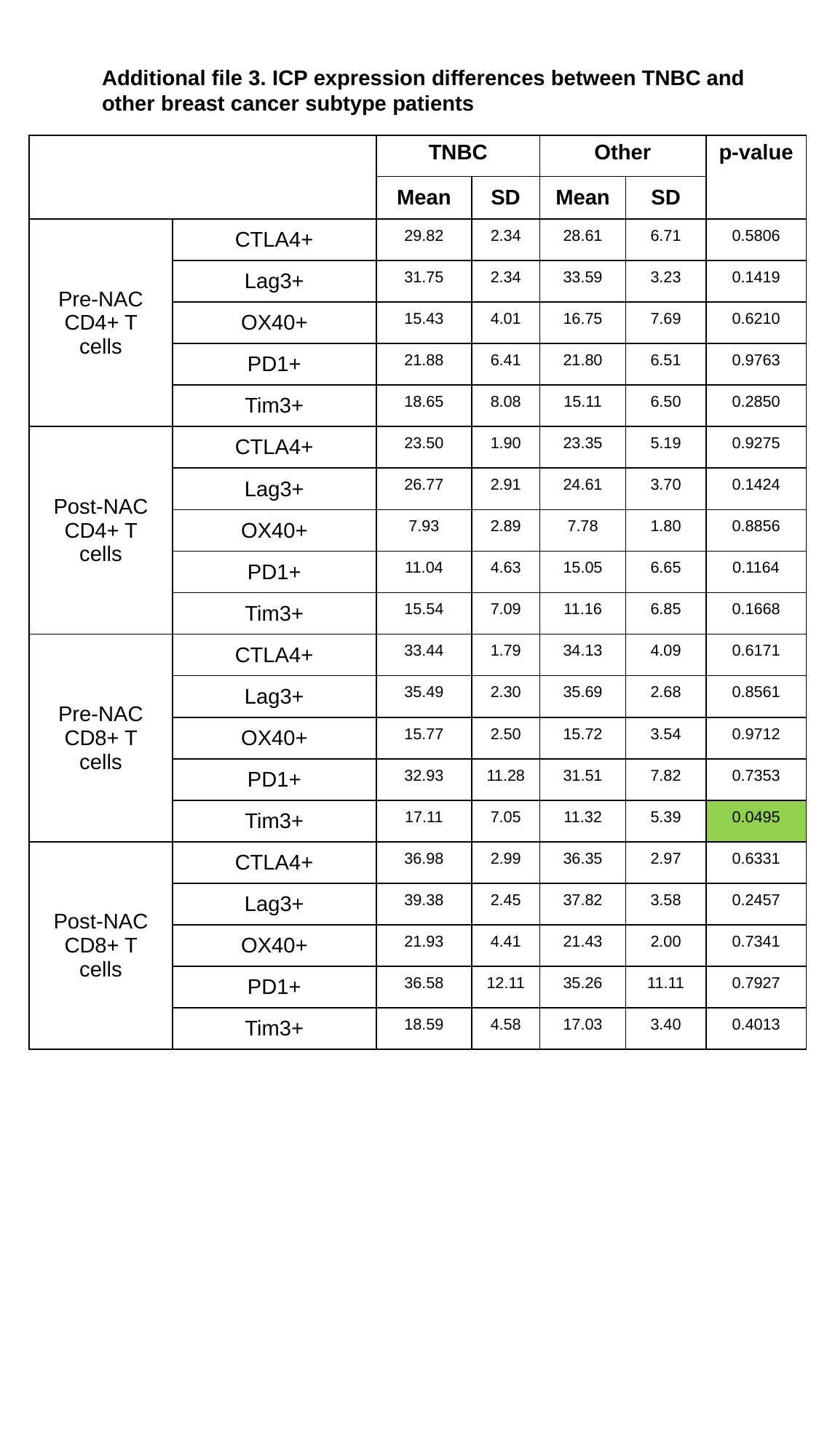

Additional file 3. ICP expression differences between TNBC and other breast cancer subtype patients
| | | TNBC | | Other | | p-value |
| --- | --- | --- | --- | --- | --- | --- |
| | | Mean | SD | Mean | SD | |
| Pre-NAC CD4+ T cells | CTLA4+ | 29.82 | 2.34 | 28.61 | 6.71 | 0.5806 |
| | Lag3+ | 31.75 | 2.34 | 33.59 | 3.23 | 0.1419 |
| | OX40+ | 15.43 | 4.01 | 16.75 | 7.69 | 0.6210 |
| | PD1+ | 21.88 | 6.41 | 21.80 | 6.51 | 0.9763 |
| | Tim3+ | 18.65 | 8.08 | 15.11 | 6.50 | 0.2850 |
| Post-NAC CD4+ T cells | CTLA4+ | 23.50 | 1.90 | 23.35 | 5.19 | 0.9275 |
| | Lag3+ | 26.77 | 2.91 | 24.61 | 3.70 | 0.1424 |
| | OX40+ | 7.93 | 2.89 | 7.78 | 1.80 | 0.8856 |
| | PD1+ | 11.04 | 4.63 | 15.05 | 6.65 | 0.1164 |
| | Tim3+ | 15.54 | 7.09 | 11.16 | 6.85 | 0.1668 |
| Pre-NAC CD8+ T cells | CTLA4+ | 33.44 | 1.79 | 34.13 | 4.09 | 0.6171 |
| | Lag3+ | 35.49 | 2.30 | 35.69 | 2.68 | 0.8561 |
| | OX40+ | 15.77 | 2.50 | 15.72 | 3.54 | 0.9712 |
| | PD1+ | 32.93 | 11.28 | 31.51 | 7.82 | 0.7353 |
| | Tim3+ | 17.11 | 7.05 | 11.32 | 5.39 | 0.0495 |
| Post-NAC CD8+ T cells | CTLA4+ | 36.98 | 2.99 | 36.35 | 2.97 | 0.6331 |
| | Lag3+ | 39.38 | 2.45 | 37.82 | 3.58 | 0.2457 |
| | OX40+ | 21.93 | 4.41 | 21.43 | 2.00 | 0.7341 |
| | PD1+ | 36.58 | 12.11 | 35.26 | 11.11 | 0.7927 |
| | Tim3+ | 18.59 | 4.58 | 17.03 | 3.40 | 0.4013 |
