# Supplementary material for: Exploratory analysis of immune checkpoint receptor expression by circulating T cells and tumor specimens in patients receiving neo-adjuvant chemotherapy for operable breast cancer
Source: BMC Cancer. 2020 May 19;20:445. doi: 10.1186/s12885-020-06949-4 (PMC7236344; doi:10.1186/s12885-020-06949-4)
Supplement: Supplementary file 4 — Additional file 4. ICP expression differences between HR positive and HR negative breast cancer patients. Pre- and post-NAC levels of CD4+ and CD8+ T cell ICP expression were compared between the HR+ and HR- breast cancer patients. Unpaired Student’s t-test was used to compare these groups. A green box indicates a statistically significant difference between HR+ and HR- breast cancer patients’ ICP expression. [file 12885_2020_6949_MOESM4_ESM.pptx]

## Slide 1
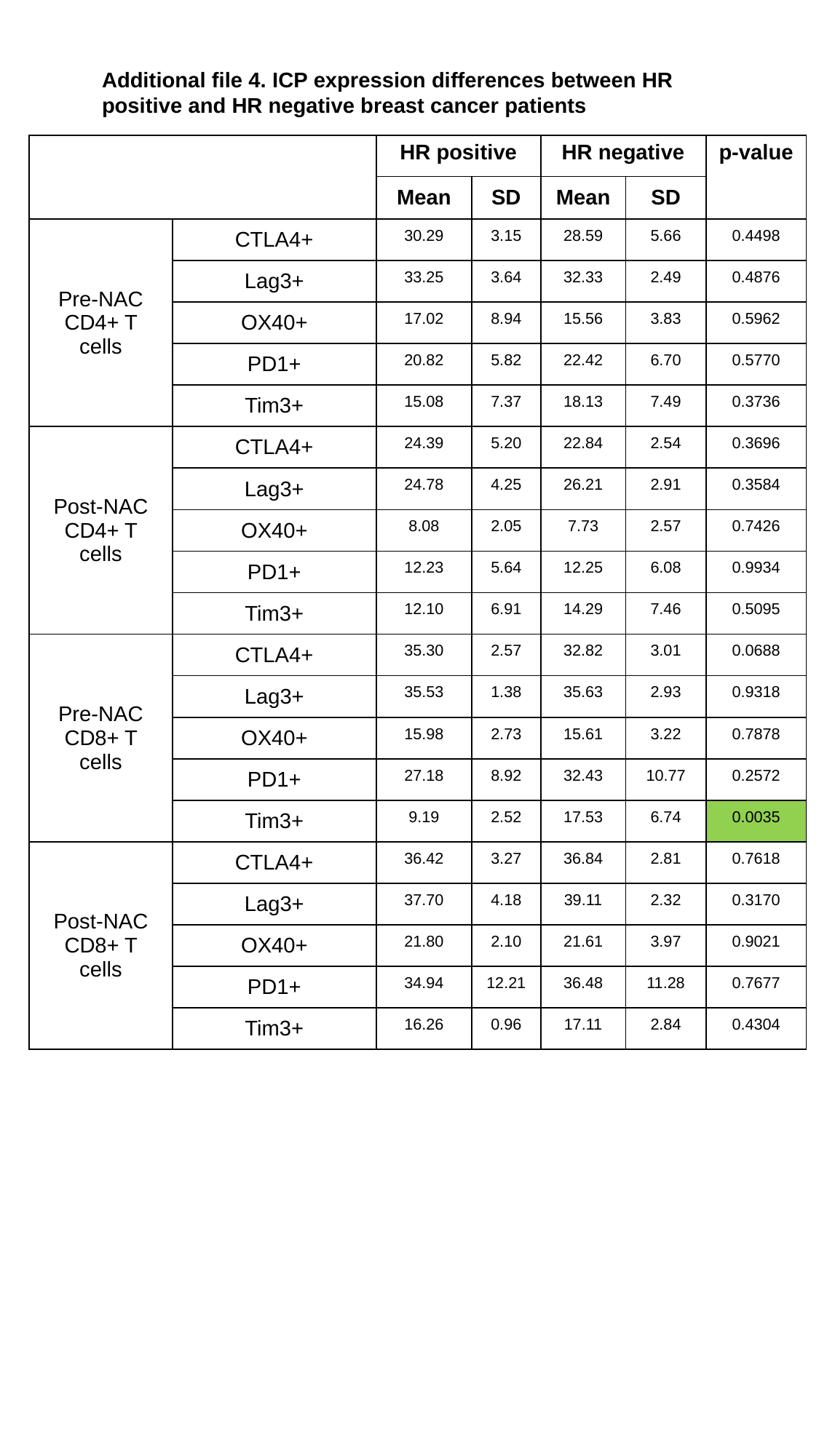

Additional file 4. ICP expression differences between HR positive and HR negative breast cancer patients
| | | HR positive | | HR negative | | p-value |
| --- | --- | --- | --- | --- | --- | --- |
| | | Mean | SD | Mean | SD | |
| Pre-NAC CD4+ T cells | CTLA4+ | 30.29 | 3.15 | 28.59 | 5.66 | 0.4498 |
| | Lag3+ | 33.25 | 3.64 | 32.33 | 2.49 | 0.4876 |
| | OX40+ | 17.02 | 8.94 | 15.56 | 3.83 | 0.5962 |
| | PD1+ | 20.82 | 5.82 | 22.42 | 6.70 | 0.5770 |
| | Tim3+ | 15.08 | 7.37 | 18.13 | 7.49 | 0.3736 |
| Post-NAC CD4+ T cells | CTLA4+ | 24.39 | 5.20 | 22.84 | 2.54 | 0.3696 |
| | Lag3+ | 24.78 | 4.25 | 26.21 | 2.91 | 0.3584 |
| | OX40+ | 8.08 | 2.05 | 7.73 | 2.57 | 0.7426 |
| | PD1+ | 12.23 | 5.64 | 12.25 | 6.08 | 0.9934 |
| | Tim3+ | 12.10 | 6.91 | 14.29 | 7.46 | 0.5095 |
| Pre-NAC CD8+ T cells | CTLA4+ | 35.30 | 2.57 | 32.82 | 3.01 | 0.0688 |
| | Lag3+ | 35.53 | 1.38 | 35.63 | 2.93 | 0.9318 |
| | OX40+ | 15.98 | 2.73 | 15.61 | 3.22 | 0.7878 |
| | PD1+ | 27.18 | 8.92 | 32.43 | 10.77 | 0.2572 |
| | Tim3+ | 9.19 | 2.52 | 17.53 | 6.74 | 0.0035 |
| Post-NAC CD8+ T cells | CTLA4+ | 36.42 | 3.27 | 36.84 | 2.81 | 0.7618 |
| | Lag3+ | 37.70 | 4.18 | 39.11 | 2.32 | 0.3170 |
| | OX40+ | 21.80 | 2.10 | 21.61 | 3.97 | 0.9021 |
| | PD1+ | 34.94 | 12.21 | 36.48 | 11.28 | 0.7677 |
| | Tim3+ | 16.26 | 0.96 | 17.11 | 2.84 | 0.4304 |
