# Supplementary material for: Exploratory analysis of immune checkpoint receptor expression by circulating T cells and tumor specimens in patients receiving neo-adjuvant chemotherapy for operable breast cancer
Source: BMC Cancer. 2020 May 19;20:445. doi: 10.1186/s12885-020-06949-4 (PMC7236344; doi:10.1186/s12885-020-06949-4)
Supplement: Supplementary file 5 — Additional file 5. Intensity of PD-L1 and PD-1 expression. Table of intensity of tissue staining for PD-L1 and PD-1. Values are denoted as means with ranges in the parentheses. [file 12885_2020_6949_MOESM5_ESM.pptx]

## Slide 1
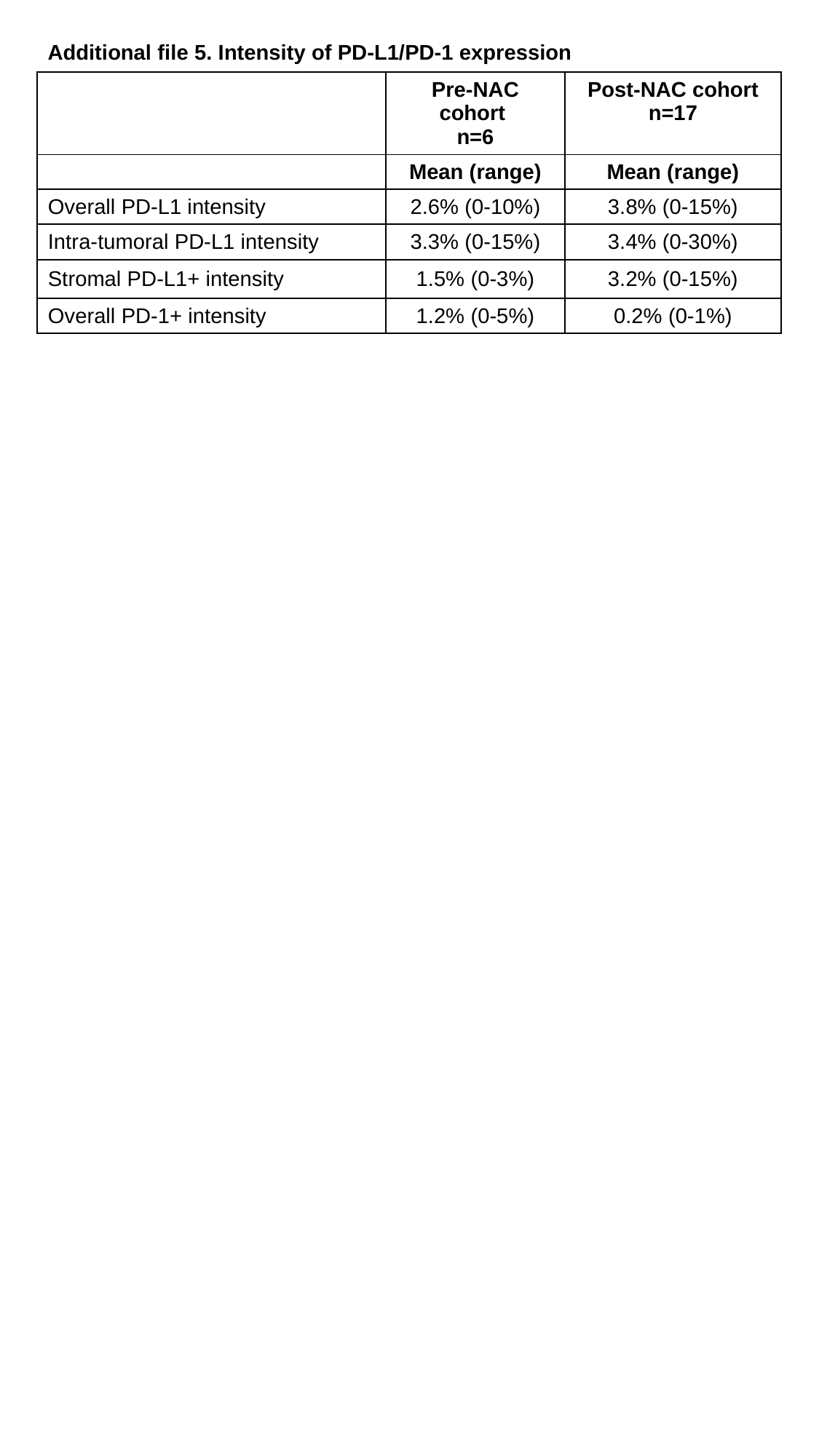

Additional file 5. Intensity of PD-L1/PD-1 expression
| | Pre-NAC cohort n=6 | Post-NAC cohort n=17 |
| --- | --- | --- |
| | Mean (range) | Mean (range) |
| Overall PD-L1 intensity | 2.6% (0-10%) | 3.8% (0-15%) |
| Intra-tumoral PD-L1 intensity | 3.3% (0-15%) | 3.4% (0-30%) |
| Stromal PD-L1+ intensity | 1.5% (0-3%) | 3.2% (0-15%) |
| Overall PD-1+ intensity | 1.2% (0-5%) | 0.2% (0-1%) |
