# Supplementary material for: Exploratory analysis of immune checkpoint receptor expression by circulating T cells and tumor specimens in patients receiving neo-adjuvant chemotherapy for operable breast cancer
Source: BMC Cancer. 2020 May 19;20:445. doi: 10.1186/s12885-020-06949-4 (PMC7236344; doi:10.1186/s12885-020-06949-4)
Supplement: Supplementary file 6 — Additional file 6. Percentage of TILs, CD8+ T cells, and PD-L1+/PD-1+ cells in TNBC samples before and after NAC. Table of changes in TILs, CD8+ T cells, PD-L1 expression and PD-1 expression following NAC in samples from triple-negative breast cancer patients. There were three pre-NAC samples available and nine post-NAC samples available for analysis. Values are listed as means (and ranges) or the number of samples (and percentage of total group these represented). If samples stained < 1%, they were considered to have 0% expression for mean calculation. PD-L1 and PD-1 positivity was defined as ≥1% expression. [file 12885_2020_6949_MOESM6_ESM.pptx]

## Slide 1
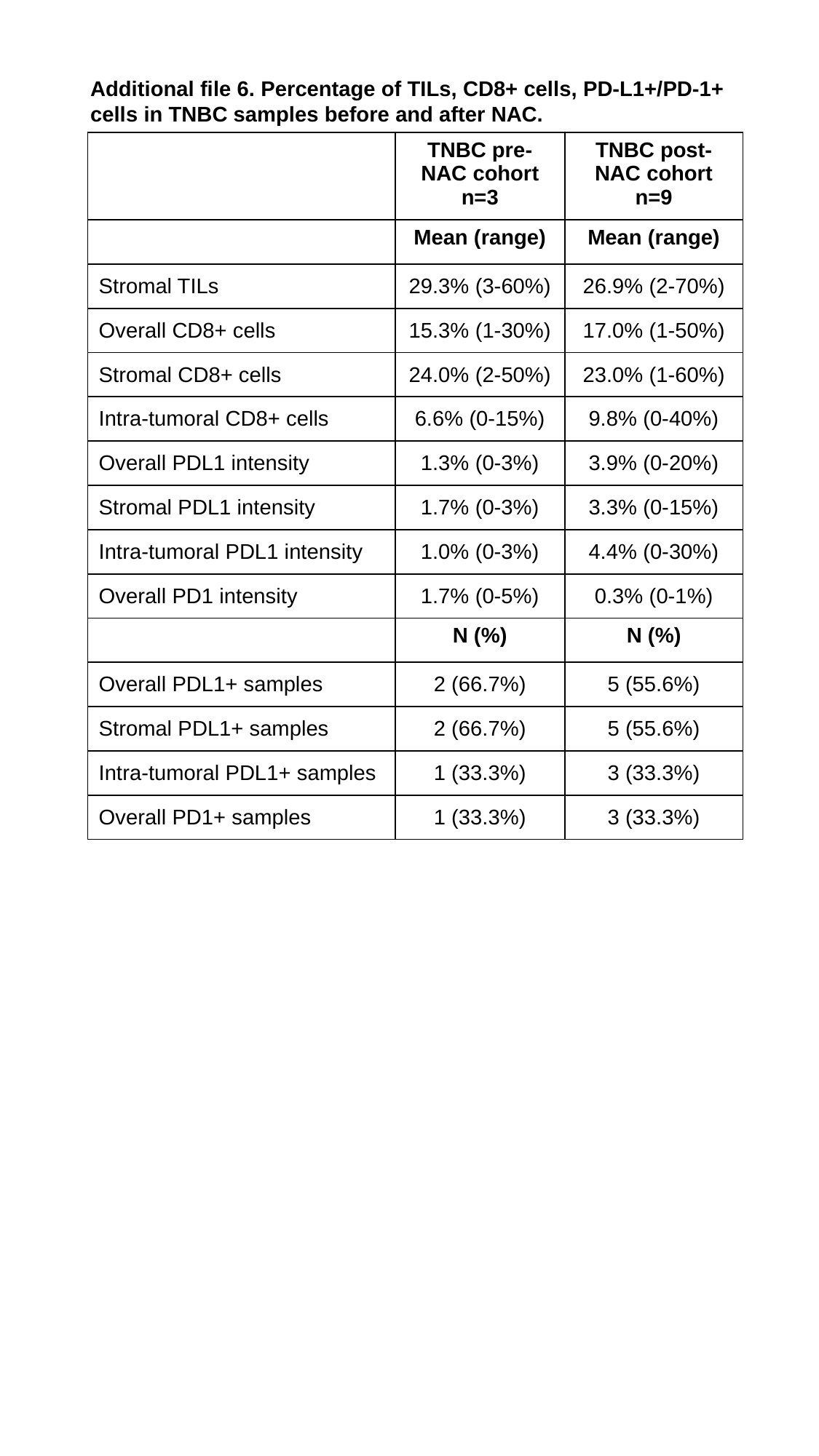

Additional file 6. Percentage of TILs, CD8+ cells, PD-L1+/PD-1+ cells in TNBC samples before and after NAC.
| | TNBC pre-NAC cohort n=3 | TNBC post-NAC cohort n=9 |
| --- | --- | --- |
| | Mean (range) | Mean (range) |
| Stromal TILs | 29.3% (3-60%) | 26.9% (2-70%) |
| Overall CD8+ cells | 15.3% (1-30%) | 17.0% (1-50%) |
| Stromal CD8+ cells | 24.0% (2-50%) | 23.0% (1-60%) |
| Intra-tumoral CD8+ cells | 6.6% (0-15%) | 9.8% (0-40%) |
| Overall PDL1 intensity | 1.3% (0-3%) | 3.9% (0-20%) |
| Stromal PDL1 intensity | 1.7% (0-3%) | 3.3% (0-15%) |
| Intra-tumoral PDL1 intensity | 1.0% (0-3%) | 4.4% (0-30%) |
| Overall PD1 intensity | 1.7% (0-5%) | 0.3% (0-1%) |
| | N (%) | N (%) |
| Overall PDL1+ samples | 2 (66.7%) | 5 (55.6%) |
| Stromal PDL1+ samples | 2 (66.7%) | 5 (55.6%) |
| Intra-tumoral PDL1+ samples | 1 (33.3%) | 3 (33.3%) |
| Overall PD1+ samples | 1 (33.3%) | 3 (33.3%) |
