# Supplementary material for: Exploratory analysis of immune checkpoint receptor expression by circulating T cells and tumor specimens in patients receiving neo-adjuvant chemotherapy for operable breast cancer
Source: BMC Cancer. 2020 May 19;20:445. doi: 10.1186/s12885-020-06949-4 (PMC7236344; doi:10.1186/s12885-020-06949-4)
Supplement: Supplementary file 7 — Additional file 7. Percentage of TILs, CD8+ T cells, and PD-L1+/PD-1+ cells in HR positive breast cancer samples before and after NAC. Table of changes in TILs, CD8+ T cells, PD-L1 expression and PD-1 expression following NAC in samples from HR positive breast cancer patients. There were two pre-NAC samples available and seven post-NAC samples available for analysis. Values are listed as means (and ranges) or the number of samples (and percentage of total group these represented). If samples stained < 1%, they were considered to have 0% expression for mean calculation. PD-L1 and PD-1 positivity was defined as ≥1% expression. [file 12885_2020_6949_MOESM7_ESM.pptx]

## Slide 1
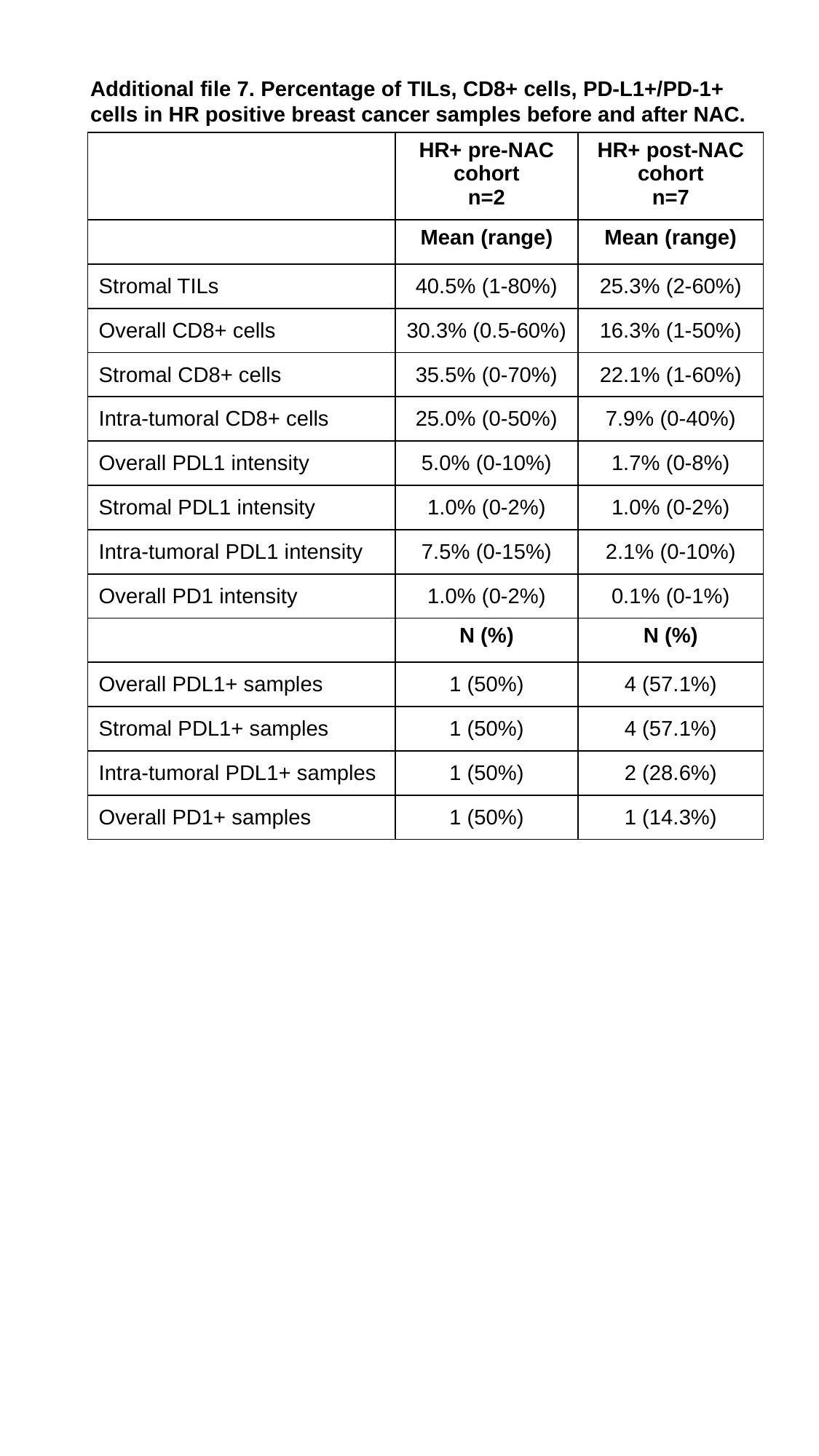

Additional file 7. Percentage of TILs, CD8+ cells, PD-L1+/PD-1+ cells in HR positive breast cancer samples before and after NAC.
| | HR+ pre-NAC cohort n=2 | HR+ post-NAC cohort n=7 |
| --- | --- | --- |
| | Mean (range) | Mean (range) |
| Stromal TILs | 40.5% (1-80%) | 25.3% (2-60%) |
| Overall CD8+ cells | 30.3% (0.5-60%) | 16.3% (1-50%) |
| Stromal CD8+ cells | 35.5% (0-70%) | 22.1% (1-60%) |
| Intra-tumoral CD8+ cells | 25.0% (0-50%) | 7.9% (0-40%) |
| Overall PDL1 intensity | 5.0% (0-10%) | 1.7% (0-8%) |
| Stromal PDL1 intensity | 1.0% (0-2%) | 1.0% (0-2%) |
| Intra-tumoral PDL1 intensity | 7.5% (0-15%) | 2.1% (0-10%) |
| Overall PD1 intensity | 1.0% (0-2%) | 0.1% (0-1%) |
| | N (%) | N (%) |
| Overall PDL1+ samples | 1 (50%) | 4 (57.1%) |
| Stromal PDL1+ samples | 1 (50%) | 4 (57.1%) |
| Intra-tumoral PDL1+ samples | 1 (50%) | 2 (28.6%) |
| Overall PD1+ samples | 1 (50%) | 1 (14.3%) |
