# Supplementary material for: Exploratory analysis of immune checkpoint receptor expression by circulating T cells and tumor specimens in patients receiving neo-adjuvant chemotherapy for operable breast cancer
Source: BMC Cancer. 2020 May 19;20:445. doi: 10.1186/s12885-020-06949-4 (PMC7236344; doi:10.1186/s12885-020-06949-4)
Supplement: Supplementary file 8 — Additional file 8. Comparison of pre- and post-NAC ICP expression in peripheral blood T cells to intra-tumoral PD-L1 expression. Colored bars show individual values of (A) CTLA, (B) Lag3, (C) OX40, (D) PD-1, and (E) Tim3 expression in pre-NAC (solid pattern) and post-NAC (striped pattern) CD4+ (blue) and CD8+ (red) T cells. Black bars reveal pre-NAC (solid pattern) and post-NAC (striped pattern) levels of intra-tumoral PD-L1 intensity; values are also listed above bars). [file 12885_2020_6949_MOESM8_ESM.pptx]

## Slide 1
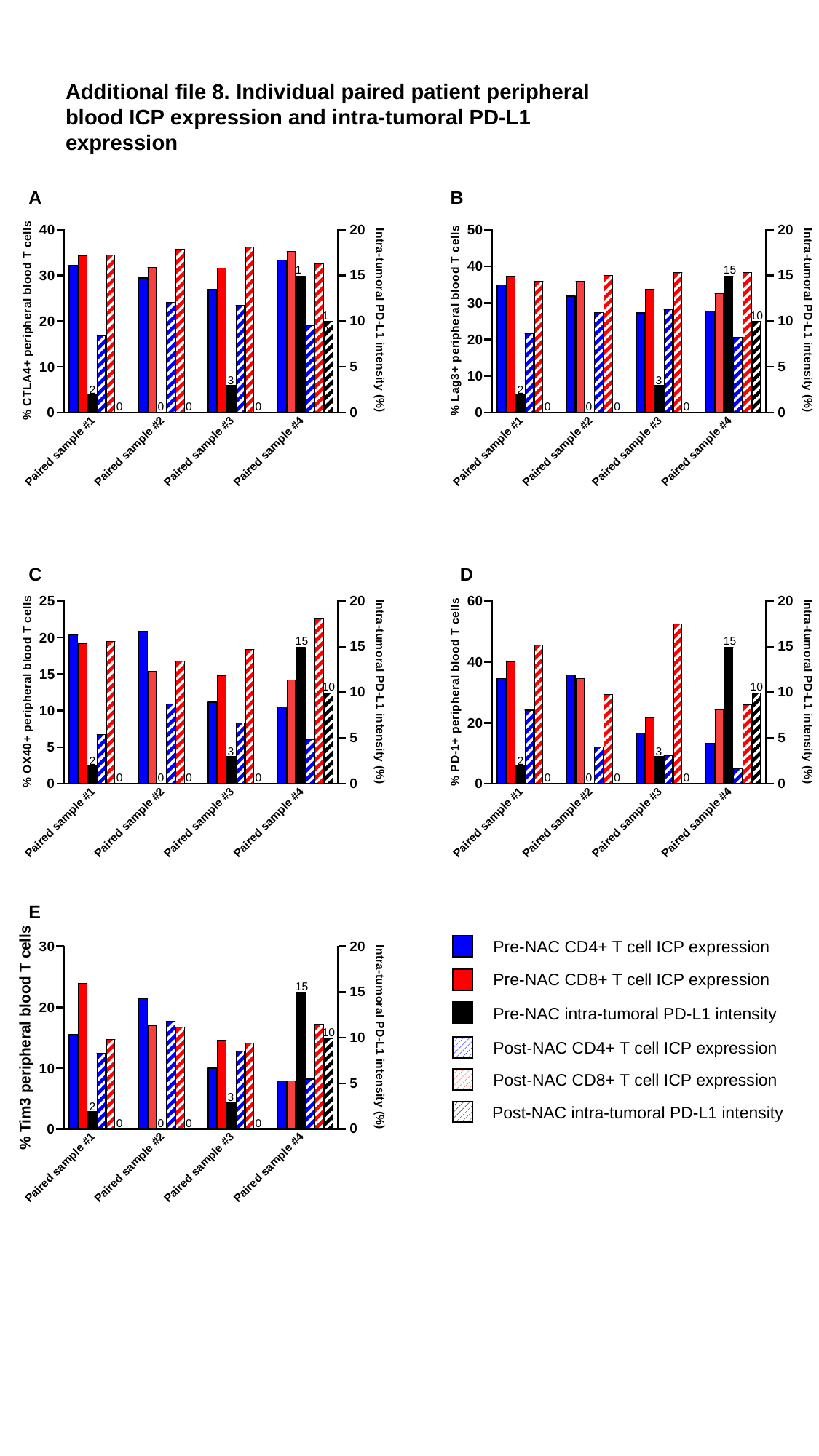

Additional file 8. Individual paired patient peripheral blood ICP expression and intra-tumoral PD-L1 expression
A
B
C
D
E
Pre-NAC CD4+ T cell ICP expression
Pre-NAC CD8+ T cell ICP expression
Pre-NAC intra-tumoral PD-L1 intensity
Post-NAC CD4+ T cell ICP expression
Post-NAC CD8+ T cell ICP expression
Post-NAC intra-tumoral PD-L1 intensity
